# 

**Published:** 1868-07-01

**Authors:** 


VOL. XXV. NUMBER 13.

### THE CHICAGO MEDICAL JOURNAL.

*PUBLISHED SEMI-MONTHLY.*

EDITED BY

J. ADAMS ALLEN, M.D., LL.D.

JULY 1, 1868.

CHICAGO:

CHURCH, GOODMAN AND DONNELLEY, STEAM BOOK AND JOB PRINTERS,

108 AND 110 DEARBORN STREET.

